# GAP-CP: Gender, Age, and Presentation in Chest Pain

**DOI:** 10.7759/cureus.90173

**Published:** 2025-08-15

**Authors:** Stephanos Ghobrial, Dimitrios Nasiakos, Nils Kinnman, Wessam Andrawes, Harry Jackson-Smith, Mohamed Elbouhi, Andreas Sarantopoulos, Johannes Luckmann, Moloy Sarkar, Karim Andrawes, Balram Chandranpillai, Laurie Fripp, Jacob Hughes, Esther Johnson, Charlotte Mcinnes, Parwathi Nair, Aryan Parajuli, Kostantinos Tasoulis, William Pickles, Annabel Rimmer, Isabella Talbot, Cristina M Thiebaud, Lorna Morse, Thisara Samarawickrama, Sen Devadathan

**Affiliations:** 1 Cardiology, Royal Cornwall Hospital NHS Trust, Truro, GBR; 2 Cardiology, European University Cyprus, Truro, GBR; 3 Acute Medicine, Royal Cornwall Hospital NHS Trust, Truro, GBR; 4 Nephrology, Royal Cornwall Hospital NHS Trust, Truro, GBR; 5 Hospital Medicine, Royal Cornwall Hospital NHS Trust, Truro, GBR; 6 Otolaryngology, Royal Cornwall Hospital NHS Trust, Truro, GBR; 7 Haematology, Royal Cornwall Hospital NHS Trust, Truro, GBR; 8 Clinical Nursing, Royal Cornwall Hospital NHS Trust, Truro, GBR

**Keywords:** age and sex differences, catheter angiogram, chest pain stratification, computed tomography coronary angiogram (ctca), coronary artery disease, invasive vs non invasive testing, rapid access chest pain clinic

## Abstract

Chest pain is a common symptom with significant diagnostic challenges, particularly as its presentation and associated outcomes can vary by sex and age. This retrospective cohort study examined 10,220 patients referred to a UK Rapid Access Chest Pain Clinic between 2018 and 2024 to explore differences in presentation, investigation pathways and coronary artery disease (CAD) severity. Patients were categorised by sex, age group and chest pain type (typical, atypical or non-cardiac), and the type of investigation or management they received was assessed. Men were more likely to report typical chest pain and undergo invasive angiography, while women more often presented atypically and were managed with non-invasive imaging. In patients with typical chest pain, men were significantly more likely to have severe CAD on angiography, especially in older age groups. These findings support risk-based models that incorporate sex and age, such as the European Society of Cardiology (ESC) risk factor-weighted clinical likelihood approach, rather than a one-size-fits-all computed tomography coronary angiography (CTCA)-first pathway recommended by the National Institute for Health and Care Excellence (NICE). The study highlights the importance of using sex- and age-specific strategies in the triage of chest pain to improve diagnostic accuracy, reduce delays, and ensure timely identification of patients at highest risk of significant coronary disease.

## Introduction

Chest pain is a common symptom that may indicate underlying coronary artery disease (CAD), a leading cause of global morbidity and mortality. Early identification and accurate diagnosis are critical for improving outcomes, especially in patients at high risk for acute coronary syndromes. Rapid Access Chest Pain Clinics (RACPCs) in the UK have become essential for the prompt triage and evaluation of patients presenting with chest pain, guiding decisions for further investigations such as computed tomography coronary angiography (CTCA) and invasive coronary angiography (ICA) [1]. While these clinics play a critical role in timely CAD diagnosis, the diagnostic process can be influenced by multiple variables, including sex, age, and the nature of chest pain presentation, with significant implications for patient outcomes.

It is well-documented that men and women experience and report chest pain differently, impacting diagnosis and management. Men commonly present with “typical” angina-substernal chest discomfort exacerbated by exertion and relieved by rest or nitroglycerin. Conversely, women are more likely to present with “atypical” symptoms such as fatigue, indigestion, nausea, or pain in other locations like the back or jaw [2]. A substantial body of evidence demonstrates that women with cardiovascular disease are often under-investigated and undertreated, contributing to sex-based disparities in mortality and morbidity [3-6]. Despite this, real-world data from UK RACPCs are limited, leaving it unclear whether the sex differences observed in broader studies are also applicable to patients attending these specialised clinics.

Current clinical guidelines offer differing approaches to chest pain assessment. The National Institute for Health and Care Excellence (NICE) guidelines (2016) recommend CTCA as the first-line investigation for most patients with stable chest pain and no known CAD, aiming to streamline diagnosis and reduce unnecessary invasive procedures [7]. Under NICE, any patient with new-onset chest pain and no known CAD is typically referred straight for CTCA unless contraindications exist.

In contrast, the 2024 European Society of Cardiology (ESC) Guidelines employ a risk factor-weighted clinical likelihood (RF-CL) model. This model integrates age, sex, chest pain characteristics, and traditional CAD risk factors (family history, smoking, dyslipidaemia, hypertension, and diabetes) to estimate the probability of obstructive CAD, an update from their previous 2019 ‘Simple Pre-Test Probability’ model which considered only age, sex, and symptoms [8].

These differing guidelines present a challenge for RACPCs. While NICE advocates for uniform CTCA usage, the ESC’s RF-CL model may guide clinicians toward different, potentially more invasive, investigation strategies based on sex, type of chest pain, and presence of risk factors. The SCOT-HEART trial [9], a landmark multicentre study involving over 4,000 patients with suspected stable angina, demonstrated that CTCA is effective in ruling out CAD and reducing unnecessary invasive testing. However, a significant proportion of patients with a higher pre-test probability - determined by factors such as sex, age, and chest pain typicality - were more likely to require subsequent ICA. This observation raises the critical question of whether a more refined risk stratification model could identify patients at greatest risk of significant CAD, allowing for earlier consideration of invasive testing such as ICA, thereby ensuring appropriate and equitable care. 

This study aims to explicitly assess how sex, age, and chest pain presentation influence diagnostic decision-making and disease severity in a UK RACPC population, with the goal of identifying potential disparities and informing optimization of clinical pathways.

## Materials and methods

**Figure 1:**
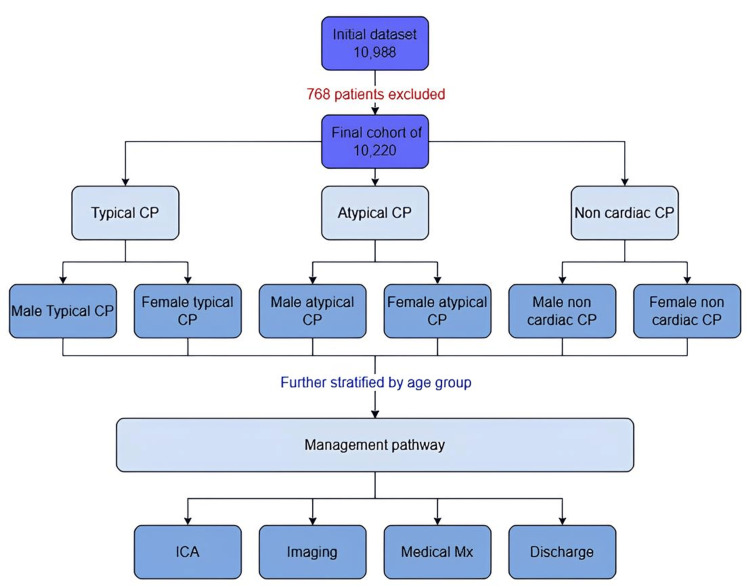
Subgroup stratification flowchart This flowchart outlines the analytical framework and patient stratification used in this study. The total patient cohort was initially stratified by chest pain type (typical, atypical, and non-cardiac), then further divided by sex (male and female), and subsequently by age group to create the subgroups used for analysis. CP: chest pain, ICA: invasive coronary angiography

This study aims to explore sex and age differences in chest pain presentation, subsequent clinical management, and disease severity in patients attending an RACPC in a District General Hospital (DGH) in Southwest England. Specifically, the study will examine sex disparities in cardiovascular care by analysing data from a large cohort of patients, seeking to determine whether men and women with chest pain are appropriately investigated and whether the existing diagnostic frameworks provide evidence-based care or require adjustments to mitigate potential biases and improve outcomes across sexes. The patient cohort was initially stratified by type of chest pain and sex, and subsequently further broken down by age. The six resulting subgroups, along with their detailed breakdown, are illustrated in Figure 1, which outlines the analytical framework used in this study.

Study design and setting

This retrospective cohort study was conducted using data from the RACPC at a large District General Hospital and primary Percutaneous Coronary Intervention centre in the Southwest England. The study utilised patient records from the hospital’s digitalised system, covering the period from 2018 to 2024. Use of anonymised data for this study was approved by the hospital’s Information Governance and Audit Department (Reference: Cardio/CA/2024-25/03). All data were fully de-identified in compliance with UK GDPR and the Data Protection Act 2018, and used solely for academic purposes.

Study population

The initial dataset comprised 10,988 patients referred to the RACPC during the study period. Patients were excluded if they had missing data on age, sex, or chest pain type or if they were admitted directly from the RACPC. Following these exclusions, a final cohort of 10,220 patients was included in the analysis.

Patients’ groups

Patients were categorised based on chest pain type using standardised diagnostic definitions consistent with the NICE guidelines [1], with which all RACPC clinicians are familiar. Typical angina was defined as substernal discomfort, provoked by exertion, and relieved by rest or glyceryl trinitrate (GTN). Atypical chest pain was defined as meeting two out of the three criteria above, while non-anginal chest pain met none or only one of the three criteria. Symptom classification was therefore standardised across clinicians, although a degree of variability inherent to patient self-report was unavoidable. Patients were further sub-grouped according to sex (male and female) and subsequently subdivided by age groups by decade (30-39, 40-49, 50-59, 60-69, ≥70) to explore differences in chest pain presentation and management across age and sex. We chose to focus on these subgroups, rather than further stratifying by traditional risk factors, in order to reduce confounding factors and provide clear outcomes. It should also be noted that although the acronym GAP originally referred to “Gender, Age and Pain type/Presentation”, all analyses were conducted using recorded biological sex (male/female) rather than self-identified gender.

Initial management pathways

For each chest pain category (typical, atypical, and non-anginal), the initial clinical management was assessed. Management pathways were classified into four categories: ICA; non-invasive imaging, including CTCA, stress magnetic resonance imaging (MRI), myocardial perfusion scan (MPS), and exercise tolerance test (ETT); medical management only; and discharge without further investigations.

Comparisons were made across the six subgroups: males and females with typical chest pain, males and females with atypical chest pain, and males and females with non-anginal pain. Each subgroup was further broken down by age to evaluate whether patterns of care varied with patient age.

Focused subgroup analysis: patients with typical chest pain

**Figure 2:**
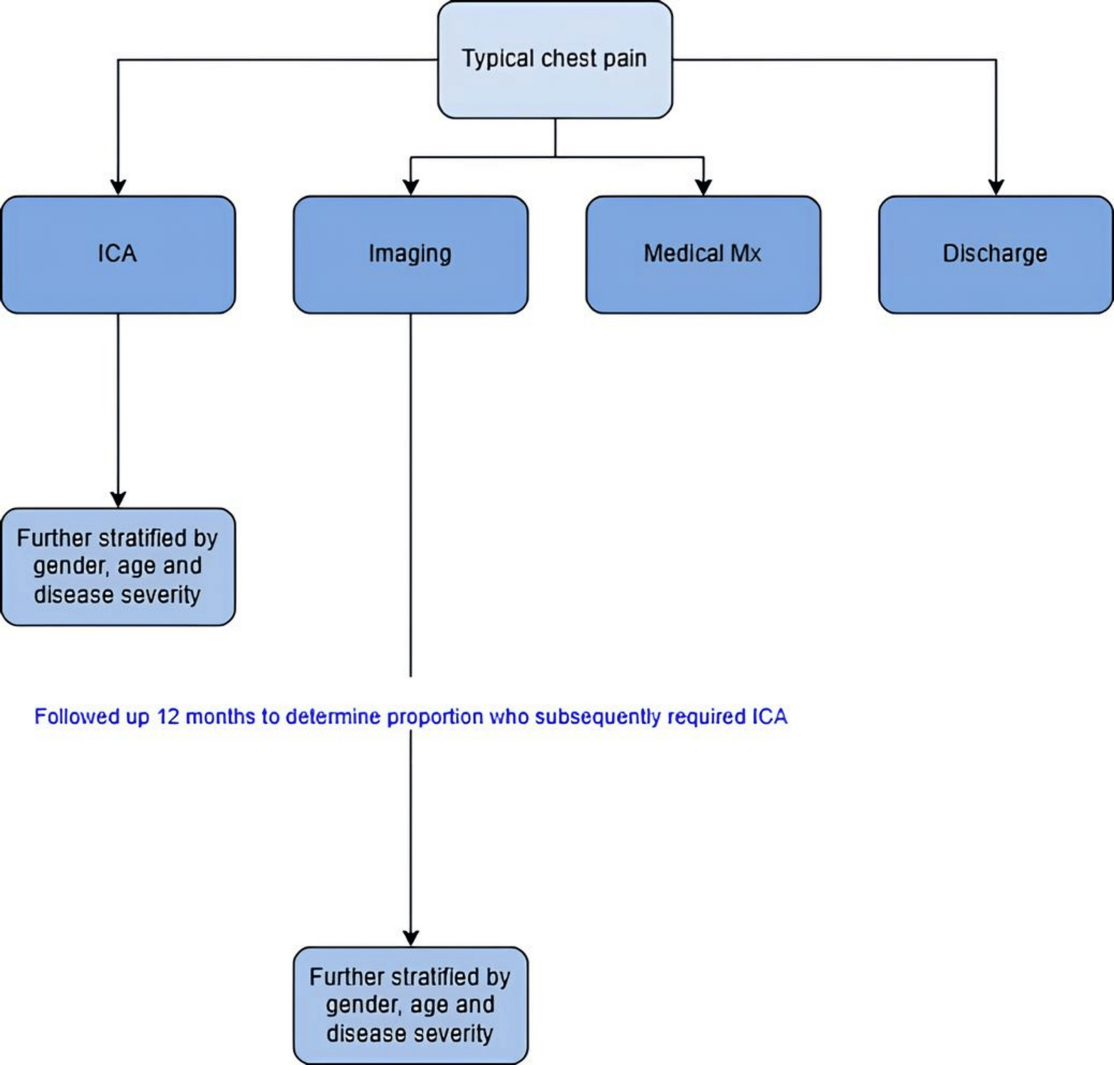
Typical chest pain management and stratification flowchart This flowchart details the management pathways for patients presenting with typical chest pain. The figure illustrates the stratification of this cohort into four primary pathways: invasive coronary angiography (ICA), non-invasive imaging, medical management, and discharge. It further outlines the subsequent assessment of disease severity within both the direct ICA group and the non-invasive imaging group, where patients were followed for up to 12 months for subsequent ICA.

A targeted analysis was conducted on patients with typical chest pain to focus on those most likely to have significant coronary artery disease requiring invasive testing, thereby minimising confounding from variable symptom profiles between sexes [10]. These patients were stratified by RACPC outcome into four pathways: medical management, discharge, non‑invasive testing and invasive testing, and each pathway was further subdivided by sex, age and disease severity. Within the invasive pathway, we assessed lesion severity in individuals referred directly for ICA and compared findings between males and females. In the non‑invasive pathway, patients were followed for up to 12 months to identify those who later required ICA, with sex, age and disease severity evaluated at the time of subsequent angiography (Figure 2).

Disease severity was classified as ‘yes’ if any lesion met the following criteria: ‘Occluded’ (100% stenosis), ‘Critical’ (>90% stenosis), or ‘Severe’ (≥70% stenosis). Additionally, left main coronary artery stenosis greater than 50% was considered significant. The terms ‘Significant’ or ‘Flow-limiting’ refer to any lesion causing hemodynamically relevant obstruction likely to impair coronary blood flow. These categories reflect commonly used clinical thresholds in routine angiographic assessment to identify lesions warranting consideration for intervention, regardless of whether intervention was performed at the time.

Statistical analysis

Chi-squared tests were used to compare the distribution of management pathways across sex, age groups, and chest pain categories. For contingency tables with expected cell counts less than five, Fisher’s exact test was employed. Categorical proportions are presented with 95% confidence intervals calculated using the Clopper-Pearson method. Where appropriate, effect sizes were quantified using Cramér’s V, with interpretation thresholds based on Cohen’s conventions (small: V=0.10, moderate: V=0.30, large: V=0.50) [11]. For comparisons involving more than two groups, pairwise post-hoc analyses with Bonferroni correction were performed. For binary proportions, absolute and relative risks with 95% confidence intervals were reported. Trends in proportions across age groups were assessed using the Cochran-Armitage test for trend. All statistical tests were two-tailed, with statistical significance set at p<0.05. Analyses were conducted using R version 4.3.2 (R Foundation for Statistical Computing, Vienna, Austria).

## Results

The data presented explores sex- and age-related differences in chest pain presentation, diagnostic pathways, and disease severity, with a specific focus on patients presenting with typical chest pain.

Chest pain presentation and sex differences

As shown in Table 1, there were clear sex-based differences in chest pain presentation types. The findings indicated that females more frequently presented with atypical chest pain (1838, 39.1%), while males showed a higher proportion of typical chest pain (2073, 37.6%). The distribution of non-anginal pain was very similar between the sexes. A Pearson’s chi-square test confirmed a highly significant association between sex and chest pain type (χ2=57.68, df=2, p<0.001), indicating a genuine difference in presentation patterns. However, the effect size was small (Cramér’s V = 0.075), suggesting that sex contributes modestly to the variability in presentation. Post-hoc pairwise χ2 tests with Bonferroni correction (adjusted significance threshold p<0.0167) were all statistically significant: typical versus atypical (χ2=57.68, df=1, p<0.001), typical versus non-anginal (χ2=15.18, df=1, p<0.001), and atypical versus non-anginal (χ2=11.04, df=1, p<0.001). These results demonstrate that the distribution of chest pain types differed significantly by sex, with females more likely to present with atypical angina and males more commonly seen with typical angina.

**Table 1:** Sex differences in chest pain presentation. The distribution of chest pain (CP) types (typical, atypical, and non-anginal) among male and female patients. The overall Pearson's chi-square test showed a significant association between sex and chest pain type (χ2=57.68, p<0.001).

Type of chest pain	Females n (%)	Males n (%)
Typical CP	1464 (31.1%)	2073 (37.6%)
Atypical CP	1838 (39.1%)	1815 (32.9%)
Non-anginal CP	1400 (29.8%)	1630 (29.5%)
Total	4702 (100.0%)	5518 (100.0%)

Sex differences in type of intervention

Typical Chest Pain

Among patients with typical chest pain (Table 2), males were substantially more likely to undergo invasive coronary angiography compared to females. Conversely, females were more frequently managed with non-invasive imaging. Medical management and discharge without further investigation were infrequent and showed minimal differences between sexes. The overall distribution of management pathways differed significantly by sex (χ2=49.64, df=3, p<0.001; Cramér’s V=0.12), reflecting a small-to-moderate effect size. Post-hoc pairwise comparisons confirmed this difference was primarily driven by the higher rate of invasive investigation in males and non-invasive imaging in females.

**Table 2:** Type of intervention by chest pain type and sex The distribution of management pathways (Invasive, Imaging, Medical, Discharged) for patients with different types of chest pain (CP), stratified by sex. The number of patients (n) and the percentage (%) are shown for each category. The χ2 and p-value for the overall chi-square analysis comparing management pathways between males and females are also included for each chest pain type.

Chest Pain Type	Sex	Invasive	Imaging	Medical	Discharged	χ2	p-value
Typical CP	Females	295 (20.2%)	1051 (71.8%)	92 (6.3%)	26 (1.8%)	49.64	<0.001
	Males	630 (30.4%)	1276 (61.6%)	140 (6.8%)	27 (1.3%)		
Atypical CP	Females	59 (3.2%)	1623 (88.3%)	69 (3.8%)	87 (4.7%)	52.46	<0.001
	Males	160 (8.8%)	1488 (82.1%)	73 (4.0%)	91 (5.0%)		
Non-anginal CP	Females	16 (1.1%)	404 (28.9%)	22 (1.6%)	958 (68.4%)	3.52	0.32
	Males	19 (1.2%)	470 (29.1%)	41 (2.5%)	1087 (67.2%)		

Atypical Chest Pain

A similar pattern of intervention was observed among patients with atypical chest pain (Table 2). Males again showed a greater propensity for invasive coronary angiography, while non-invasive imaging remained the predominant management strategy for females. Medical management and discharge were rare for both sexes. The sex difference in management strategy was statistically significant (χ2=52.46, df=3, p<0.001; Cramér’s V=0.12). Post-hoc pairwise comparisons with Bonferroni correction indicated that males were significantly more likely to undergo invasive investigation compared to each of the other management strategies (all adjusted p<0.001), while no significant differences were found among the other pairwise comparisons (all adjusted p>0.05).

Non-anginal Chest Pain

For non-anginal chest pain (Table 2), the management approach was largely consistent across sexes. Most patients, regardless of sex, were discharged without further investigation or managed with non-invasive imaging. Invasive investigation and medical management were rarely employed for either sex. There was no significant sex difference in the overall distribution of management pathways (χ2=3.52, df=3, p=0.32; Cramér’s V=0.03), indicating a negligible effect size, and no post-hoc pairwise comparisons reached statistical significance.

Age‐subdivided management pathways in chest pain presentations

Typical Chest Pain

Analysis of management pathways for typical chest pain patients revealed a significant trend with age. The use of invasive angiography rose sharply with advancing age, climbing from virtually absent in younger patients (30-39 years) to becoming a substantial pathway for males aged 70 and above, though non-invasive imaging remained the most common approach for this age group. Over the same span, the use of non-invasive imaging fell, transitioning from the predominant pathway in younger adults to being used in just over half of patients in the oldest age group (Table 3). Medical management and discharge were infrequent overall but became modestly more common in older patients.

**Table 3:** Management pathway for patients presenting with typical chest pain, subdivided by age and sex The distribution of management pathways (Invasive, Imaging, Medical, Discharged) for patients with Typical Chest Pain, stratified by sex and age group. The number of patients (n) and the percentage (%) are shown for each category. The χ2 and p-value for the chi-square analysis comparing males and females within each age group are included

Age Group	Sex	Invasive	Imaging	Medical	Discharged	Total n	χ2	p-value
30-39	Males	1 (7.1%)	13 (92.9%)	0 (0.0%)	0 (0.0%)	14	-	1
	Females	0 (0.0%)	9 (100.0%)	0 (0.0%)	0 (0.0%)	9		
40-49	Males	11 (12.1%)	75 (82.4%)	4 (4.4%)	1 (1.1%)	91	3.41	0.33
	Females	10 (13.3%)	64 (85.3%)	0 (0.0%)	1 (1.3%)	75		
50-59	Males	85 (22.7%)	280 (74.7%)	8 (2.1%)	2 (0.5%)	375	1.86	0.6
	Females	47 (18.4%)	203 (79.3%)	5 (2.0%)	1 (0.4%)	256		
60-69	Males	177 (27.5%)	438 (68.1%)	21 (3.3%)	7 (1.1%)	643	18.42	<0.001
	Females	76 (17.2%)	347 (78.5%)	10 (2.3%)	9 (2.0%)	442		
70+	Males	352 (37.2%)	470 (49.7%)	107 (11.3%)	17 (1.8%)	946	36.61	<0.001
	Females	158 (23.3%)	428 (63.1%)	77 (11.4%)	15 (2.2%)	678		

When stratified by age, no significant sex differences in management pathways were observed among patients aged 30-39 (Fisher’s exact test, p=1.00), 40-49 (χ2=3.41, df=3, p=0.33; Cramér’s V=0.14), or 50-59 (χ2=1.86, df=3, p=0.60; Cramér’s V=0.05).

However, significant sex differences emerged among older patients. In the 60-69 age group, males were more likely to receive invasive management compared to females, with a significant difference in the overall distribution of management strategies (χ2=18.42, df=3, p<0.001; Cramér’s V=0.13). This disparity was even more pronounced in patients aged 70 and above (χ2=36.61, df=3, p<0.001; Cramér’s V=0.15).

A Cochran-Armitage trend test further confirmed a significant increase in the use of invasive management with advancing age for both men (Z=7.23, p<0.001) and women (Z=3.13, p=0.0018), though the magnitude of the trend was greater among men.

These findings indicate that sex-based differences in management of typical chest pain are most apparent in older age groups, with men increasingly more likely to undergo invasive testing than women as age advances.

Atypical Chest Pain 

Analysis of management pathways for atypical chest pain patients showed distinct trends with age and sex. As detailed in Table 4, invasive testing remained a rare pathway for all age groups and both sexes. The use of non-invasive imaging, while the most common approach, declined with age, while pharmacological management and discharge increased in frequency, particularly in older patients..

**Table 4:** Management pathway for patients presenting with atypical chest pain, subdivided by age and sex The distribution of management pathways (Invasive, Imaging, Medical, Discharged) for patients with atypical chest pain, stratified by sex and age group. The number of patients (n) and the percentage (%) are shown for each category. The χ2 and p-value for the chi-square analysis comparing males and females within each age group are included

Age Group	Sex	Invasive	Imaging	Medical	Discharged	Total n	χ2	p-value
30-39	Males	0 (0.0%)	51 (94.4%)	0 (0.0%)	3 (5.6%)	54	-	0.037
	Females	0 (0.0%)	241 (88.0%)	3 (1.1%)	30 (10.9%)	274		
40-49	Males	8 (3.9%)	192 (92.8%)	3 (1.4%)	4 (1.9%)	207	2.67	0.45
	Females	7 (4.2%)	147 (89.1%)	3 (1.8%)	8 (4.8%)	165		
50-59	Males	43 (8.3%)	447 (86.3%)	9 (1.7%)	19 (3.7%)	518	17.32	<0.001
	Females	14 (2.9%)	450 (93.9%)	4 (0.8%)	11 (2.3%)	479		
60-69	Males	50 (9.2%)	446 (82.1%)	24 (4.4%)	23 (4.2%)	543	32.41	<0.001
	Females	12 (2.1%)	518 (92.0%)	12 (2.1%)	21 (3.7%)	563		
70+	Males	59 (12.0%)	352 (71.8%)	37 (7.6%)	42 (8.6%)	490	24.58	<0.001
	Females	25 (4.2%)	480 (80.5%)	47 (7.9%)	44 (7.4%)	596		

When stratified by age, sex differences in management pathways for atypical chest pain were not significant in most younger groups. Among patients aged 30-39, a significant difference in management distribution was observed (Fisher’s exact test, p=0.037), although both sample size and event rates were low in this cohort. For patients aged 40-49, no significant sex difference in management distribution was found (χ2=2.67, df=3, p=0.45; Cramér’s V=0.08).

Significant sex differences emerged in older age groups. Among those aged 50-59, the distribution of management strategies differed by sex (χ2=17.32, df=3, p<0.001; Cramér’s V=0.13), with men more likely to receive invasive management and women more often managed with non-invasive imaging. The disparity was even more marked in patients aged 60-69 (χ2=32.41, df=3, p<0.001; Cramér’s V=0.17) and in those aged 70 and above (χ2=24.58, df=3, p<0.001; Cramér’s V=0.15), again reflecting a higher likelihood of invasive and medical management in men.

A Cochran-Armitage trend test confirmed a significant increase in the use of invasive management with advancing age among men (Z=4.08, p<0.001), but not among women (Z=0.85, p=0.39), highlighting a sex-based difference in the management escalation for atypical chest pain.

These findings indicate that sex-based differences in the management of atypical chest pain become significant from midlife onwards, with men increasingly likely to undergo invasive procedures as age advances, whereas women are more often managed non-invasively across all ages.

Non-anginal Chest Pain

As detailed in Table 5, the management of non-anginal chest pain was characterized by a clear shift towards discharge with advancing age. Discharge without further testing was the predominant pathway, rising from 57 (61.3%) of males aged 30-39 to 354 (76.5%) of females in the oldest cohort. Concurrently, the use of non-invasive imaging decreased with age, while invasive angiography remained a negligible pathway at all ages.

**Table 5:** Management pathway for patients presenting with non-anginal chest pain, subdivided by age and sex The distribution of management pathways (Invasive, Imaging, Medical, Discharged) for patients with non-anginal chest pain, stratified by sex and age group. The number of patients (n) and the percentage (%) are shown for each category. A Fisher’s exact test was performed for all age groups, with the p-value for the comparison between males and females included.

Age Group	Sex	Invasive	Imaging	Medical	Discharged	Total n	p-value
30-39	Males	0 (0.0%)	35 (37.6%)	1 (1.1%)	57 (61.3%)	93	1
	Females	0 (0.0%)	18 (36.7%)	1 (2.0%)	30 (61.2%)		
40-49	Males	2 (0.8%)	84 (33.3%)	3 (1.2%)	163 (64.7%)	252	0.79
	Females	0 (0.0%)	56 (35.2%)	1 (0.6%)	102 (64.2%)		
50-59	Males	4 (0.9%)	146 (32.1%)	8 (1.8%)	297 (65.3%)	455	0.16
	Females	8 (2.2%)	130 (36.1%)	3 (0.8%)	219 (60.8%)		
60-69	Males	5 (1.3%)	132 (33.2%)	8 (2.0%)	252 (63.5%)	397	0.33
	Females	4 (1.1%)	108 (29.6%)	3 (0.8%)	250 (68.5%)		
70+	Males	8 (1.9%)	73 (17.4%)	21 (5.0%)	318 (75.7%)	420	0.21
	Females	4 (0.9%)	91 (19.7%)	14 (3.0%)	354 (76.5%)		

When stratified by age, no significant sex differences were observed in the distribution of management strategies for non-anginal chest pain. In all age groups, the comparison of management pathways between men and women was performed using Fisher’s exact test due to low event counts in several categories. The results were not statistically significant in any decade: 30-39 years, p=1.00; 40-49 years, p=0.79; 50-59 years, p=0.16; 60-69 years, p=0.33; and 70 years and older, p=0.21.

A Cochran-Armitage trend test demonstrated no significant increase in the use of invasive management with advancing age in either men (Z=1.86, p=0.063) or women (Z=0.03, p=0.97).

These findings indicate that, in contrast to typical and atypical chest pain, sex-based differences in management strategies for non-anginal chest pain are not apparent across age groups, and the likelihood of invasive testing does not increase substantially with age in either sex.

Typical chest pain cohort

Disease Severity Differences Between Males and Females With Age Sub-groups

Among patients presenting with typical chest pain, substantial sex differences in disease severity were observed, with men consistently exhibiting higher rates of significant coronary artery disease across all pathways. This trend generally persisted across age groups, older patients demonstrated a greater burden of disease, though it was not uniformly consistent.

Imaging Followed by Subsequent ICA Within 12 Months

As detailed in Table 6, among patients with typical chest pain who underwent non-invasive imaging followed by invasive coronary angiography within 12 months, the proportion with severe coronary disease increased with age in both sexes. Overall, men were significantly more likely to have severe disease (298/463, 64.4%, 95% CI: 59.8-68.7%) compared to women (97/203, 47.8%, CI: 40.7-54.9%). This difference was statistically significant (χ2=15.3, p<0.001), with males exhibiting a 35% higher relative risk (RR=1.35) of severe disease.

**Table 6:** Imaging Followed by Subsequent ICA within 12 months for Typical Chest Pain The number of patients who underwent invasive coronary angiography (ICA) after non-invasive imaging, and the number and percentage of those found to have severe coronary disease. The data is stratified by sex and age. The χ2 and p-value for the comparison of severe disease rates between males and females are included.

Age	Sex	n ICA	n Severe	% Severe	χ2	p-value
30-39	Females	1	0	0	-	1
	Males	3	1	33.3		
40-49	Females	3	0	0	-	0.21
	Males	18	10	55.6		
50-59	Females	30	16	53.3	0.04	0.85
	Males	78	45	57.7		
60-69	Females	74	32	43.2	11.4	0.001
	Males	169	108	63.9		
70+	Females	95	49	51.6	6.4	0.01
	Males	195	134	68.7		
TOTAL	Females	203	97	47.8	15.3	<0.001
	Males	463	298	64.4		

When stratified by age group, no significant sex difference was observed among patients aged 30-39, 40-49, or 50-59 (p>0.05 for all), as the confidence intervals for the proportion of females and males with severe disease overlapped in these younger cohorts.

However, significant differences emerged in older age groups, with men consistently showing a higher proportion of severe disease than women. This disparity became more pronounced with advancing age, as demonstrated by the divergence of the confidence intervals in the 60+ cohorts. For example, the difference was significant in patients aged 60-69 (χ2=11.4, p=0.001; Cramér’s V=0.19) and in those aged 70 and above (χ2=6.4, p=0.01; Cramér’s V=0.17).

These findings demonstrate that men are significantly more likely than women to have severe disease on invasive angiography, a disparity that is most apparent in older age groups.

Direct ICA Cohort

Among patients with typical chest pain who proceeded directly to invasive coronary angiography, the prevalence of severe coronary disease was substantially higher in males than in females across most age groups. Overall, 22.8% of females (58/254; 95% CI: 17.8-28.5%) and 54.6% of males (293/537; 95% CI: 50.2-58.8%) were found to have severe disease (Table 7).

**Table 7:** Prevalence of severe disease found in patient with typical chest pain who went direct to ICA, subdivided by age and sex The number of patients with typical chest pain who proceeded directly to invasive coronary angiography (ICA), and the number and percentage of those found to have severe coronary disease. The data is stratified by sex and age. The χ2 and p-value for the comparison of severe disease rates between males and females are included.

Age	Sex	n ICA	n Severe	% Severe	χ2	p-value
30-39	Females	0	0	0	-	-
	Males	1	0	0		
40-49	Females	9	1	11.1	-	1
	Males	9	2	22.2		
50-59	Females	41	3	7.3	-	0.002
	Males	74	22	29.7		
60-69	Females	66	20	30.3	5.7	0.02
	Males	148	71	48		
70+	Females	138	34	24.6	44.2	<0.001
	Males	305	198	64.9		
TOTAL	Females	254	58	22.8	66.7	<0.001
	Males	537	293	54.6		

The absolute risk difference was 31.7%, corresponding to a relative risk of 2.39 for males versus females. This sex difference in severe disease rates was highly significant (χ2=66.7, df=1, p<0.001; Cramér’s V=0.30).

Age-stratified analyses revealed no significant sex difference in the youngest cohorts, though this was partly due to low event rates in females. However, significant disparities were observed from the 50-59 group onwards, with the prevalence of severe disease being significantly higher in males than in females (Fisher’s exact test, p=0.002). This disparity became even more pronounced with advancing age, as the 95% confidence intervals for the proportion of severe disease, which overlapped in the youngest age groups, diverged significantly in the 60+ cohorts. For example, in the 70+ age group, the difference was highly significant (χ2=44.2, p<0.001).

These findings demonstrate that, in the direct ICA cohort, men with typical chest pain are more than twice as likely as women to have severe coronary disease, particularly among those aged 50 years and older.

All ICA Combined

Among all patients with typical chest pain who underwent invasive coronary angiography, males had a substantially higher prevalence of severe coronary disease compared to females across all age groups (Table 8). Overall, men were significantly more likely to have severe disease (591/1000, 59.1%; 95% CI: 56.0-62.2) than women (155/457, 33.9%; 95% CI: 29.6-38.5), a difference that was highly significant (χ2=79.0, p<0.001; Cramér’s V=0.23). The absolute risk difference between sexes was 25.2%, corresponding to a relative risk of 1.74 for males.

**Table 8:** All ICA Cases for Typical Chest Pain The combined data for all patients with typical chest pain who underwent invasive coronary angiography (ICA), regardless of whether it was done directly or after non-invasive imaging. The table shows the total number of patients, the number and percentage with severe coronary disease, and the statistical comparison of severe disease rates between males and females.

Age	Sex	n ICA	n Severe	% Severe	χ2	p-value
30-39	Females	1	0	0	-	1
	Males	4	1	25		
40-49	Females	12	1	8.3	-	0.03
	Males	27	12	44.4		
50-59	Females	71	19	26.8	5.5	0.02
	Males	152	67	44.1		
60-69	Females	140	52	37.1	13.8	<0.001
	Males	317	179	56.5		
70+	Females	233	83	35.6	61.8	<0.001
	Males	500	332	66.4		
TOTAL	Females	457	155	33.9	79	<0.001
	Males	1000	591	59.1		

Age-stratified analyses revealed a clear pattern of widening sex disparity with age. While severe disease was observed in both men and women across all age groups, significant differences in the prevalence of severe disease emerged from the 40-49 age group onwards (Fisher’s exact test, p=0.03). This disparity became more pronounced with each successive decade. For example, in the 70+ age group, 66.4% of males (332/500) had severe disease compared to 35.6% of females (83/233), a highly significant difference (χ2=61.8, p<0.001; Cramér’s V=0.29).

The 95% confidence intervals for the proportion of severe disease overlapped in the youngest age groups but diverged with increasing age, reflecting a widening sex disparity in severe angiographic disease.

These findings indicate that, among all patients undergoing ICA for typical chest pain, males are significantly more likely than females to have severe coronary disease, with the greatest disparities observed in older age groups.

## Discussion

The findings of this study show the significant sex and age disparities in chest pain presentation, subsequent management, and disease severity within a Rapid Access Chest Pain Clinic in a southwest DGH.

Chest pain presentation and initial intervention

A clear sex difference was observed in the presentation of chest pain, where atypical pain was significantly higher in women than men. These findings are consistent with previous literature indicating that women more often report non-classical symptoms [2].

It has previously been documented that, within cardiovascular research, female participants are significantly understudied and this inadequate enrolment and reporting limits our ability to introduce sex or gender specific guidelines [3,4,6]. Our participants were 46% women and as such, our data provides valuable insights into sex differences in presentation and management of chest pain. We found that the type of intervention differed notably by both sex and symptom typicality. The fact that males (with typical or atypical chest pain) were more likely to undergo invasive coronary angiography as an initial investigative procedure than females may reflect both gender-based bias in clinical decision-making and/or genuine differences in disease presentation or risk. Without data on the mortality and morbidity of our cohort, it is difficult to confidently unpick the implications of the observed disparity in investigation and management, but our results follow previously observed trends of more invasive investigation in men and raise questions about the equity of our current standards of care.

Sex and age disparity in disease severity

Our results demonstrate a significant sex disparity in the severity of CAD among patients undergoing ICA, which is consistent with prior studies indicating that men tend to develop obstructive CAD at higher rates [12]. In our cohort, a greater number of men were referred directly for ICA and, of the total number who underwent the procedure, men were nearly twice as likely as women to have severe disease. This finding, which persisted across all age groups, indicates male sex as an independent risk factor for anatomically significant CAD in those presenting with typical chest pain. Age was also a strong determinant of both investigation type and disease severity. Among men with typical chest pain, the rate of invasive angiography was considerably higher in those over 70 compared to younger cohorts, and women followed a similar pattern. This escalation reflects adherence to risk stratification principles. The severity of CAD increased with age across both sexes, which is in keeping with the cumulative nature of atherosclerosis and the higher burden of cardiovascular risk factors in older individuals. The observed trends in our study align with the ESC's RF-CL model, which stratifies males as being at higher risk for obstructive CAD and incorporates age as a key determinant. This reinforces the need for sex- and age-adjusted diagnostic strategies, and highlights the importance of considering both factors in risk-based stratification models to guide clinical decision-making.

Implications for RACPC diagnostic framework

Contrary to NICE recommendations, which advocate for CTCA as the first-line investigation, a notable proportion of both male and female patients in this cohort were referred directly from RACPC to ICA without prior non-invasive imaging. This deviation from guideline-based pathways likely reflects the clinical expertise of RACPC nurses working closely with cardiology consultants.

The divergence between NICE guidelines and the ESC approach, particularly the use of the RF-CL model, raises important questions regarding the most effective and equitable diagnostic pathways, and may influence clinical decision-making regarding the investigation of coronary artery disease. NICE guidelines advocate for CTCA as the first-line investigation for stable chest pain, whereas ESC guidelines incorporate gender- and age-specific adjustments together with the traditional risk factors in their RF-CL model. Our data support the use of such a model which emphasises the necessity for clinicians to consider not only the presenting symptoms but also sex- and age-related nuances that may influence the risk profiles of patients.

Delays in investigation and diagnosis

NICE guidelines for stable angina emphasise timely CTCA without setting a specific timeframe, focusing on rapid diagnosis and management [1]. This vagueness is seen as a shortfall, with UK wait times varying widely, often lasting weeks or months due to limited resources [13]. Ideally, CTCA should occur promptly for early risk assessment, with groups like the British Society of Cardiovascular Imaging suggesting a timeframe of within three weeks [14], though NICE guidelines do not enforce this. The guidelines prioritise diagnostic strategy over logistics, allowing local systems to interpret “prompt” based on capacity, resulting in inconsistent application across the UK. Effective outcomes depend on quick CTCA access and rapid ICA scheduling, as delays may overlook significant CAD, highlighting the need for efficient diagnostic processes.

In the Trust where the study was conducted, the average wait time for CTCA is approximately six to eight weeks, based on data from the trust’s radiology records and RACPC logs. CTCA availability may be affected by scanner access, staffing, and patient volume, contributing to the extended wait time of eight weeks.

A subset of patients was referred directly for ICA based on clinical suspicion, supported by the clinical expertise of the chest pain nurses and the input of cardiology consultants. While NICE recommends CTCA as the first-line investigation and recognises age and gender as risk factors for clinical suspicion of angina, ESC incorporates these variables in their RF-CL, allowing clinicians to bypass CTCA and proceed directly to ICA in patients deemed high risk.

Delays in CTCA across healthcare systems globally hinder timely diagnosis of CAD, with wait times often spanning weeks to months due to resource constraints. Such delays critically affect patients with severe CAD, where prompt intervention is essential. A study on Routine Revascularisation Versus Initial Medical Therapy for Stable Ischemic Heart Disease, affiliated with the ISCHAEMIA trial [15] linked delayed revascularisation to worse outcomes. A 2015 SCOT-HEART analysis [9] further suggests early imaging reduces events, emphasising the need for expedited diagnostic pathways.

Study limitations

This study is not without limitations. As a retrospective, single-center study, our findings may not be generalisable to other populations or institutions, as variations in local clinical practice, patient demographics, and resource availability could influence outcomes. The classification of chest pain as typical, atypical, or non-anginal was based on clinician-documented symptoms, which introduces a degree of subjectivity and potential for self-report bias. Our analysis also lacks data on patient mortality and long-term morbidity such as major adverse cardiovascular events (MACE), preventing us from drawing definitive conclusions about whether the observed sex- and age-based disparities in management translated into differences in clinical outcomes. Furthermore, while we analysed key variables, our retrospective design limited the ability to account for all potential confounding factors, such as socioeconomic status or other comorbidities, which may have influenced both the clinical decision-making process and disease severity. The analysis of subsequent ICA was limited to a 12-month timeframe, which may not capture all relevant follow-up procedures. Future prospective multicenter studies with longer follow-up periods are needed to confirm our findings and assess their impact on patient outcomes.

## Conclusions

This study shows that within our cohort of patients attending RACPC, there were clear sex and age differences observed in chest pain presentation, subsequent clinical management and disease severity. Severity of coronary disease increased with age across both sexes. Females were significantly more likely to present atypically and be investigated using non-invasive imaging. Conversely, men were observed to present more commonly with typical chest pain and were also more likely to undergo invasive testing. In our subgroup analysis of typical chest pain, men were more likely to have significant disease demonstrated on ICA, either as the initial investigation or after initial imaging. This evidence supports the ESC RF-CL model, which stratifies male sex and advancing age as higher risk for obstructive CAD, over the current NICE guidance which recommends CTCA as first line. Risk stratification by age, sex and chest pain typicality has the potential to streamline the diagnostic process and reduce delays for those patients who are most likely to have clinically significant CAD. This is particularly relevant in a world of ever-increasing wait times due to resource constraints. While our findings provide important insights, further prospective studies are warranted to confirm their impact on mortality and morbidity outcomes.
